# Professor Max Sänger

**Published:** 1903-03

**Authors:** 


Professor Max Sänger, of Prague, died at his home, January 12, 1903, aged 50 years. This distinguished gynecologist was born at Bayreuth in 1853, and began his professional career in 1878, when he was appointed assistant to Credé. Professor Sänger was the author of a monograph on Cesarean section, for which he devised a special method of suturing the uterus. He also wrote about 150 articles on tumors, plastic operations, abdominal section, retrodisplacements, and other subjects. He was a pioneer of vaginal hysterectomy in Germany, performing the operation for the first time in 1881, and he was a strong advocate of aseptic and antiseptic methods in gynecological and obstetric surgery. In 1899 he succeeded Rosthorn in the chair of gynecology at the University of Prague. He was an honorary Fellow of the American Association of Obstetricians and Gynecologists.

